# Students perspective on the impact of gender on medical training and future practice

**DOI:** 10.2147/AMEP.S131856

**Published:** 2017-02-08

**Authors:** Rahul Rajesh Amin, Prashant Bamania

**Affiliations:** Faculty of Medicine, Imperial College London, London, UK

**Dear editor**

We read with great interest the study by Van Wyk et al^1^ which explores the beliefs and opinions held by final-year medical students on the impact of gender on their training and the medical profession they were about to enter. As fifth year medical students ourselves, studying at Imperial College London, UK, this was a study we could strongly relate to and as such, we were particularly impressed by some of the findings.

Having just finished our Obstetrics and Gynaecology (OBG) rotation, our experiences highlighted to us the issue of how one’s gender affects the training received during medical school. We agree with the findings of the study that there are obstacles to males performing intimate examinations, and even consultations, on female patients.^1^ This especially holds true in a multicultural city like London, where many different social, religious, and cultural beliefs exist. What is particularly noteworthy, however, is that we found that our gender, and status as a medical student, was the biggest contributor to a female patient’s reluctance to having a male student examine them, especially during our OBG rotation. This is in contrast to the study’s findings that the male student’s age was a greater influence than their gender on a patient’s decision.^1^ This difference from our experience may be due to cultural beliefs held in countries like South Africa, where this study was conducted, in comparison to the UK. We also noticed that the cultural background of a patient affects whether we, as male students, could examine or even talk to some female patients. Female patients, particularly the elderly, who were of a different cultural background to us were less likely to consent to male students performing basic clinical examinations on them or taking a history. Yanikkerem et al^2^ supported the notion that some women may prefer being examined by female doctors and commented that religious and cultural beliefs were among the influences for a patient’s preference.

The finding that females in particular struggled with elderly male patients who had a different cultural background to them also drew our attention.^1^ From our own experiences on the wards, we have seen how the “incompetent female” idea mentioned by Van Wyk et al^1^ is sparsely present in some elderly male and female patients who resent being treated by females. Again, this may be due to a cultural belief system, an idea echoed in a study by Adudu and Adudu^3^ who found that some patients preferred male doctors because patients thought they were more hardworking, had superior knowledge, and greater experience.

Van Wyk et al^1^ also found that some female students mentioned that an advantage of being female enables them to have a better rapport with the patient. From our experience this holds true especially in OBG where our female colleagues were able to build a better rapport by being more gentle, understanding and sympathetic. This may lead to better patient–doctor interaction and a meta-analytic review by Hall and Roter^4^ found that patients of female doctors were less anxious.

In conclusion, we agree with many findings of the study regarding experiencing obstacles during training, which often hold true for both female and male students. In an ideal world, there would be an equal number of female and male doctors in each specialty, and all patients would be equally happy being treated by any doctor regardless of gender. However, in reality, altering people’s perceptions of female doctors and changing some of their cultural and religious preferences regarding their doctor’s gender may be difficult to achieve.
